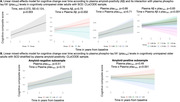# Longitudinal cognitive changes in SCD participants in relation to plasma amyloid status and the potential additive effect of plasma ptau181 levels: A CLoCODE study

**DOI:** 10.1002/alz.086729

**Published:** 2025-01-09

**Authors:** Kai Shao, Xiaochen Hu, Katharina Buerger, Emrah Düzel, Klaus Fliessbach, Beiqi He, Xueyan Jiang, Luca Kleineidam, Christoph Laske, Ruixian Li, Robert Perneczky, Oliver Peters, Josef Priller, Anja Schneider, Annika Spottke, Stefan Teipel, Xuanqian Wang, Min Wei, Yongzhe Wei, Jens Wiltfang, Jie Yang, Xianfeng Yu, Mingkai Zhang, Liang Zhang, Frank Jessen, Michael Wagner, Elizabeth Kuhn, Ying Han, DELCODE study group and SILCODE study group

**Affiliations:** ^1^ XuanWu Hospital of Capital Medical University, Beijing China; ^2^ German Center for Neurodegenerative Diseases (DZNE), Bonn Germany; ^3^ Department of Psychiatry, University of Cologne, Medical Faculty, Cologne Germany; ^4^ German Center for Neurodegenerative Diseases (DZNE), Bonn/Cologne Germany; ^5^ Institute for Stroke and Dementia Research (ISD), University Hospital, LMU, Munich Germany; ^6^ German Center for Neurodegenerative Diseases (DZNE), Munich Germany; ^7^ German Center for Neurodegenerative Diseases (DZNE), Magdeburg Germany; ^8^ Institute of Cognitive Neurology and Dementia Research (IKND), Otto‐von‐Guericke University, Magdeburg Germany; ^9^ Department of Neurodegenerative Diseases and Geriatric Psychiatry, University of Bonn Medical Center, Bonn Germany; ^10^ School of Information and Communication Engineering, Hainan University, Haikou China; ^11^ State key laboratory of digital medical engineering, School of Biomedical Engineering, Hainan University, Sanya China; ^12^ University of Bonn Medical Center, Dept. of Neurodegenerative Disease and Geriatric Psychiatry/Psychiatry, Bonn Germany; ^13^ Section for Dementia Research, Hertie Institute for Clinical Brain Research and Department of Psychiatry and Psychotherapy, University of Tuebingen, Tuebingen Germany; ^14^ German Center for Neurodegenerative Diseases (DZNE), Tübingen Germany; ^15^ LMU University Hospital, Munich Germany; ^16^ Munich Cluster for Systems Neurology (SyNergy), Munich Germany; ^17^ Department of Psychiatry and Psychotherapy, University Hospital, LMU Munich, Munich Germany; ^18^ Department of Psychiatry and Psychotherapy, Charité – Universitätsmedizin Berlin, corporate member of Freie Universität Berlin and Humboldt‐Universität zu Berlin‐Institute of Psychiatry and Psychotherapy, Berlin Germany; ^19^ German Center for Neurodegenerative Diseases (DZNE), Berlin Germany; ^20^ Department of Psychiatry and Psychotherapy, Technical University of Munich, Munich Germany; ^21^ University of Edinburgh and UK DRI, Edinburgh UK; ^22^ Department of Neurology, University of Bonn, Bonn Germany; ^23^ German Center for Neurodegenerative Diseases (DZNE), Rostock Germany; ^24^ Department of Psychosomatic Medicine, Rostock University Medical Center, Rostock Germany; ^25^ Department of Psychiatry and Psychotherapy, University Medical Center, University of Goettingen, Goettingen Germany; ^26^ German Center for Neurodegenerative Diseases (DZNE) Goettingen, Goettingen China; ^27^ Neurosciences and Signaling Group, Institute of Biomedicine (iBiMED), Department of Medical Sciences, University of Aveiro, Aveiro Portugal; ^28^ Excellence Cluster on Cellular Stress Responses in Aging‐Associated Diseases (CECAD), University of Cologne, Cologne Germany; ^29^ National Clinical Research Center for Geriatric Disorders, Beijing China; ^30^ Center of Alzheimer’s Disease, Beijing Institute for Brain Disorders, Beijing China; ^31^ Institute of Biomedical Engineering, Shenzhen Bay Laboratory, Shenzhen China; ^32^ School of Biomedical Engineering, Hainan University, Haikou China

## Abstract

**Background:**

Subjective cognitive decline (SCD), in the absence of objective cognitive impairment, may be the first symptomatic manifestation of Alzheimer's disease (AD). Previous studies have suggested that its combination with amyloid‐positivity (Aβ+) may represent stage 2 AD, and is associated with a higher risk of future cognitive decline. Here, we aim to (1) confirm this using the plasma Aβ42/40 ratio, and (2) test whether the addition of plasma phospho‐tau181 (ptau_181_, a marker of Aβ and tau pathology) could help refine the prediction of future cognitive decline in SCD patients.

**Method:**

We included 290 cognitively unimpaired older adults with SCD from two cohorts included in the ongoing Sino‐German CLoCODE collaborative project (49.3% female, 70.80±6.18y): 31 from SILCODE, and 259 from DELCODE. All participants had available baseline plasma biomarkers (Aβ42/40 ratio and ptau_181_ using the SIMOA method), and serial assessment of global cognition using a z‐transformed standard composite score over up to 6 years. Linear mixed‐effects models were conducted to confirm the predictive value of plasma Aβ+ for longitudinal cognitive decline in participants with SCD (model 1), and to determine the additive value of plasma ptau_181_ levels by examining their interaction (model 2), but also its effect in samples stratified by Aβ+/‐ (models 3).

**Result:**

In the combined CLoCODE sample, we found a significant interaction between time and Aβ confirming that Aβ+ SCD (42.1% of CLoCODE SCD patients) had a faster cognitive decline than Aβ‐ SCD patients, who showed test‐repetition improvement (p<0.001). This association remained significant when ptau_181_ was added to the model (p=0.002). A triple interaction between time, Aβ, and ptau_181_ was also found (p<0.001), showing that those with a faster cognitive decline were Aβ+ SCD patients with higher baseline ptau_181_ levels (Figure A). This was also confirmed in stratified analyses, where there was a significant interaction between time and ptau_181_ levels only in Aβ+ participants (p<0.001; Aβ‐, p=0.72; Figure B). Similar results were obtained in stratified cohort analyses.

**Conclusion:**

Our findings suggest that plasma biomarkers of AD pathology may help to identify SCD patients who will experience future cognitive decline due to AD, and thus require closer follow‐up.